# Allogenic platelet-rich plasma for treatment of knee and hip osteoarthritis

**DOI:** 10.3389/fpain.2023.1216190

**Published:** 2023-06-15

**Authors:** Ashim Gupta, Anish G. Potty, Nicola Maffulli

**Affiliations:** ^1^ Regenerative Orthopaedics, Noida, India; ^2^ Future Biologics, Lawrenceville, GA, United States; ^3^ BioIntegrate, Lawrenceville, GA, United States; ^4^ South Texas Orthopaedic Research Institute (STORI Inc.), Laredo, TX, United States; ^5^ Department of Musculoskeletal Disorders, School of Medicine and Surgery, University of Salerno, Fisciano, Italy; ^6^ San Giovanni di Dio e Ruggi D’Aragona Hospital “Clinica Orthopedica” Department, Hospital of Salerno, Salerno, Italy; ^7^ Barts and the London School of Medicine and Dentistry, Centre for Sports and Exercise Medicine, Queen Mary University of London, London, United Kingdom; ^8^ School of Pharmacy and Bioengineering, Keele University School of Medicine, Stoke-on-Trent, United Kingdom

**Keywords:** platelet-rich plasma, PRP, allogenic PRP, umbilical cord blood, osteoarthritis, knee osteoarthritis, hip osteoarthritis, regenerative medicine

## Abstract

Osteoarthritis (OA) induces tremendous amounts of stress and financial burden on patients and healthcare systems worldwide. Current treatments have limitations and do not address the etiopathogenetic cause of OA. Regenerative medicine may circumvent limitations posed by traditional modalities and relies on the utilization of biologics including platelet-rich plasma (PRP). Several peer-reviewed studies have documented the safety and efficacy of autologous PRP in mitigating symptoms in knee and hip OA patients. Nonetheless, only few studies investigated the safety and efficacy of allogenic PRP. This mini review summarizes the outcomes of preclinical and clinical studies using allogenic PRP for treatment of knee or hip OA. We identified 3 preclinical and 1 clinical study using allogenic PRP for treatment of knee OA, and only 1 clinical study using allogenic PRP for treatment of hip OA. Administration of allogenic PRP is safe and probably efficacious in patients with knee or hip OA. However, more pre-clinical studies and high-powered, multi-center, non-randomized and randomized controlled trials with extended follow-up are warranted to further establish the safety and efficacy of allogenic PRP to justify its clinical use.

## 1. Introduction

Osteoarthritis (OA) is the most prevalent joint ailment affecting millions of people throughout the world (1). OA is a degenerative joint disease normally affecting larger weight-bearing joints, including knees and hips (1). OA involves regressive changes in the articular cartilage leading to considerable pain and progressive functional limitation, thereby impacting patients’ overall quality of life (1). OA is classically managed with physiotherapy, activity modification, pharmacological agents including non-steroidal anti-inflammatory drugs (NSAIDs) and intra-articular injection of hyaluronic acid (i.e., viscosupplementation) or corticosteroids, and surgical interventions (joint arthroplasty) after conservative modalities have been ineffective (2). These conventional options have shortcomings, aiming to reduce pain but not targeting the underlying pathology (1–3).

There has been a marked increase in the use of biologics, including platelet-rich plasma (PRP), for regenerative medicine applications, especially in the field of orthopaedics (4). Multiple studies including non-randomized and randomized controlled trials, review articles, and systematic review and meta-analyses have demonstrated the safety and efficacy of autologous PRP for OA (4). On the other hand, its efficacy has been questioned for subpar outcomes, which can be attributed to patient related factors including age, comorbidities, quality and quantity of platelets, concomitant medications taken, and the lack of standardization of PRP formulation protocol (5–8). To overcome the limitations posed by autologous PRP, the potential of allogenic PRP, including PRP derived from umbilical cord blood, has been explored in patients with symptomatic knee or hip OA. Allogenic PRP derived from cord blood possesses a high content of regenerative factors, thereby making it an ideal source for allogenic PRP (9). Allogenic PRP, including umbilical cord blood-derived PRP, from well-characterized donors following standardized formulation protocol and cryopreservation methodology, can provide a viable alternative for patients suffering with knee or hip OA (9). However, to date, only limited studies have investigated the safety and efficacy of allogenic PRP for knee or hip OA. In this mini review, we summarized the outcomes of preclinical and clinical studies using allogenic PRP for knee or hip OA.

## 2. Allogenic PRP and knee osteoarthritis

### 2.1. Pre-clinical studies

Kanwat et al. (10) investigated the effect of intra-articular allogenic PRP in a Dunkin-Hartley guinea pig model of knee OA. 24 male guinea pigs weighing 600–700 g were assigned in equal numbers to two groups. In group I, one knee was used for intervention (PRP injection) and the contralateral knee as control (isotonic saline injection). Group II was similar to group I and used to determine reproducibility of results from group I. Allogenic PRP was obtained via cardiac puncture from donor animals, and 4 ml of PRP were prepared. The mean platelet count (MPC) in the whole blood was 565,000/µl and 612,000/µl for groups I and II, respectively. The MPC in the PRP was 1,820,000/µl and 1,963,000/µl for groups I and II (∼3times of baseline values), respectively. 4 ml activated PRP (activated by CaCl_2_) and 4 ml saline were administered intra-articularly in the intervention and contralateral knee, respectively. 6 animals/group were euthanized at 3 months, and the rest at 6 months post-injection for further analysis (synovial fluid cartilage oligomeric matrix protein, COMP, level analysis by ELISA and histopathological valuation of articular cartilage and synovium). The mean COMP was significantly (*p* < 0.05) lower in the knees treated with PRP compared to controls at 3 months. The mean synovitis score and synovial vascularity were lower in the knees treated with PRP compared to control at both 3 and 6 months. The mean articular cartilage degeneration was significantly lower in PRP treated knees compared to the control knees in group I only. This preliminary preclinical study demonstrated the possibility of utilizing allogenic PRP for treating knee OA, with potential anti-inflammatory and disease modifying effect, as evidenced by the chondroprotective result obtained in the PRP injected knees.

Catarino et al. (11) evaluated the impact of allogenic PRP on pain and joint function in canines with OA, refractory to conventional treatments including physical rehabilitation and pharmacological agents. This case series included 5 dogs (varying breeds, aged 6–12 year old and both genders) with OA in the elbow, tibiotarsal, knee and intercarpal joints. The allogenic PRP utilized consisted of at least 1,000,000 platelets/µl and was administered intra-articularly. The animals were evaluated at baseline and at 30, 60 and 90 days post-treatment for lameness at walk and trot (five grades—grade 1–5) and pain (five scores—grade 0–4). The results showed improvement in lameness grade at 90 days compared to baseline. Similarly, results for pain evaluation showed no pain in 3 animals and reduction from severe or moderate pain to mild pain in the remaining 2 animals at 90 days. A major limitation of this study is the lack of description of PRP formulation methodology, thereby preventing the replicability and reproducibility of this study. Despite this, the study demonstrates the potential applicability and safety of allogenic PRP for the treatment of osteoarthritic canines.

Wang et al. (12) compared the efficacy of allogenic PRP with autologous PRP on cartilage damage in a knee OA rabbit model. Healthy New Zealand rabbits (*n* = 32) were randomly assigned to 4 groups (8 rabbits/group)—control (normal knee), model (Knee OA) group, autologous PRP and allogenic PRP. The control and model groups did not receive any treatment. The treatment groups received 0.5 ml of either autologous or allogenic PRP into the knee joint once every 2 weeks for 3 times. The platelets concentration in PRP was (1,987 ± 219.48) × 10^9^/L, leukocytes was (0.14 ± 0.08) × 10^9^/L, and the RBC concentration was (0.1 ± 0.05) × 10^9^/L. Two weeks after final injection, the animals were euthanized, and the knee was further analyzed via histopathology, immunohistochemistry and gross examination. The macroscopic rating of articular cartilage in the autologous PRP group significantly improved compared to the model group, and no changes were observed compared to the control group. No significant differences were observed between the allogenic PRP group and model group. Overall, macroscopic ratings were significantly better for the autologous PRP group compared to the allogenic PRP group. Similar outcomes were observed at histopathological examination. Immunohistochemistry results showed higher BMP-2 and SOX-9 expression for both groups compared to the model group, with significantly higher expression of BMP-2 in the autologous PRP group compared to the allogenic PRP group. No difference was observed for SOX-9 expression between autologous and allogenic PRP groups. The authors concluded that autologous PRP was superior to allogenic PRP, but this study has several limitations, including the small sample size and very short follow-up duration. In spite of this, it is the first study that attempted to directly compare the efficacy of autologous PRP with allogenic PRP. More studies with higher sample size and longer follow-up are required to compare the efficacy between the two groups.

### 2.2. Clinical studies

Caiaffa et al. (13) investigated the safety and efficacy of allogenic PRP derived from umbilical cord blood in patients suffering from knee OA. Cord blood units collected at blood banks (total nucleated cell counts <1.5 × 10^9^/L, platelet count 150 × 10^9^/L and volume >50 ml) were processed to formulate allogenic PRP with platelet concentration of 800–1,200 × 10^9^/L. Patients aged 43–79 years old, with Grade I-III OA (on Kellgren-Lawrence grading scale), without acute meniscal tears were included in this study. Patients with condylar or tibial plateau bone marrow oedema (evaluated via MRI), major axial deviation (valgus, >10° or varus >5°) of the involved leg, intra-articular administration of steroid or hyaluronic acid in the last 6 months, ipsilateral ankle or hip arthritis, or preceding malignancy, were excluded from the study. All eligible patients (*n* = 25; 10 males and 15 females) received a single intra-articular injection of 10 ml allogenic umbilical cord blood-derived PRP. Patients were assessed at baseline and at 4, 8, and 12 weeks, and 6 months post-injection using different clinical outcome measures including VAS, KOOS, WOMAC and IKDC scores. No serious adverse effects were observed during the whole study. There were statistically significant (*p* < 0.05) improvements for all clinical outcome measures at each follow-up time-point compared to baseline. The main shortcomings of this study were the short duration of follow-up and lack of a control group. Nevertheless, the study demonstrated the potential of umbilical cord blood-derived allogenic PRP in knee OA patients.

## 3. Allogenic PRP and hip osteoarthritis

### 3.1. Pre-clinical studies

To date, there are no published preclinical studies involving the use of allogenic PRP for treatment of hip OA.

### 3.2. Clinical studies

Mazzotta et al. (14) investigated the safety and efficacy of umbilical cord PRP compared to autologous PRP in hip OA patients. The mean platelet concentration was 1,000  ×  10^9^/L ± 20% (4–5 times of baseline) for both autologous and umbilical cord PRP. The inclusion criteria were: patients 18–65 years old; unilateral hip pain and functional impairment for at least 4 months, with pain intensity of at least 20 on VAS (100 mm scale); BMI < 35; failure of conservative treatment; and low or intermediate OA (Tonnis 1–3) within a month prior to treatment. Exclusion criteria were: patients <18 or >65 years old; unable to provide informed consent; systemic disorders; neoplastic or local infectious problems; OA secondary to protrusio acetabuli or an excessive deformity; OA secondary to rheumatoid arthritis; local skin lesions; and pregnant women. 100 patients met the aforementioned inclusion and exclusion criteria and were included in the study. 3 weekly ultrasound guided injections of either 5 ml activated (using 10% calcium gluconate) autologous PRP (*n* = 50) or 5 ml activated (using 10% calcium gluconate) umbilical cord PRP (*n* = 50) were administered. All patients were evaluated using HHS, WOMAC and VAS scores at baseline and at follow-up time-points of 2, 6 and 12 months. 4 patients from umbilical cord PRP group did not complete the follow-up and were disqualified from the study. No major adverse events were observed during the length of the study. Both groups showed non statistically significant improvements for all clinical outcome measures at all follow-up time-points compared to baseline. Umbilical cord PRP showed statistically significant improvement for VAS and HHS at 2 months compared to baseline. However, this significant improvement was not observed at 6 and 12 months. No differences were reported between umbilical cord PRP and autologous PRP for any clinical outcome measure at all follow-up time-points. Intriguingly, sub-group analysis showed that patients with lower OA grade (Tonnis 1 or 2) experienced significant improvements in HHS with umbilical cord PRP compared to autologous PRP at 12 months. The study demonstrated that administration of umbilical cord PRP is safe. Despite no differences between umbilical cord PRP and autologous PRP, more studies are needed to determine whether it will be beneficial to use umbilical cord PRP in low grade hip OA patients based on sub-group analysis results.

## 4. Conclusion

Despite evident methodological constraints, the aforementioned preclinical and clinical studies showed that administration of allogenic PRP including umbilical cord blood-derived PRP is safe and potentially efficacious in patients with knee or hip OA. However, given the scarcity of the relevant literature, more pre-clinical studies along with high-powered, multi-centre, non-randomized and randomized controlled trials with longer follow-up are needed to further evaluate the efficacy of allogenic PRP and compare its efficacy to autologous PRP in knee and hip OA patients.

As of 2nd May 2023, there are no clinical trials registered on clinicaltrials.gov to either evaluate the safety and efficacy of allogenic PRP or compare it to autologous PRP for treatment of knee or hip OA.
